# Re-examination of the type materials of seven species of Plexippina (Araneae, Salticidae) from China

**DOI:** 10.3897/zookeys.1293.203377

**Published:** 2026-09-30

**Authors:** Yuchen Zhou, Xianjin Peng

**Affiliations:** 1 College of Life Sciences, Hunan Normal University, Changsha, Hunan 410081, China College of Life Sciences, Hunan Normal University Changsha China

**Keywords:** New synonym, Plexippini, redescription, taxonomy

## Abstract

The type specimens of seven species of the subtribe Plexippina from China are re-examined, and detailed illustrations of the habitus and copulatory organs are provided. Three species retain their current taxonomy: *Evarcha wulingensis* Peng, Xie & Kim, 1993, *Pancorius goulufengensis* Peng, Yin, Yan & Kim, 1998, *Thyene yuxiensis* Xie & Peng, 1995. The other four species have taxonomic changes and are to be treated as synonyms as follows: *Telamonia luxiensis* (Peng, Yin, Yan & Kim, 1998), **syn. nov**. (= *Epeus bicuspidatus* (Song, Gu & Chen, 1988)); *Thyene radialis* Xie & Peng, 1995 was previously synonymised with *Thyene orientalis* Żabka, 1985; *Thyene triangula* Xie & Peng, 1995, **syn. nov**. (= *Ptocasius montiformis* Song, 1991); *Thyene zhangi* (Peng, Yin, Yan & Kim, 1998), **syn. nov**. (= *Thyene orientalis* Żabka, 1985).

## Introduction

The jumping-spider tribe Plexippini represents a large and morphologically diverse lineage within the family Salticidae. The subtribe Plexippina Simon, 1901, currently comprises over 600 species in 37 genera (Marathe et al. 2024b; Wang et al. 2025; WSC 2026). Plexippina is predominantly distributed throughout the Old World and includes several well-known genera, such as *Epeus* G.W. Peckham & E.G. Peckham, 1886, *Evarcha* Simon, 1902, *Hyllus* C.L. Koch, 1846, *Pancorius* Simon, 1902, *Plexippus* C.L. Koch, 1846, *Ptocasius* Simon, 1901, *Telamonia* Thorell, 1887, *Thyene* Simon, 1885, and *Yaginumaella* Prószyński, 1979 (Maddison 2015; Cao et al. 2016; Marathe et al. 2024; Hill 2025; Wang et al. 2025). Most members of this subtribe are relatively large-bodied salticids characterised by robust legs, a generally rounded palpal bulb, and a simple, fixed embolus in males; in females, the epigynal guide is often shifted anteriorly from the posterior margin and usually divided into two lateral pockets (Maddison 2015; Logunov 2021b; Marathe et al. 2024b; Hoang 2025; Wang et al. 2025).

However, morphological conservatism, including relatively simple genital structures, pose significant challenges for delimitation and taxonomic identification of species within Plexippina (Maddison 2015; Marathe et al. 2024a, 2024b; Wang et al. 2025; WSC 2026). Recent advances in phylogenomics, particularly ultra-conserved elements (UCEs), have significantly improved our understanding of the relationships within Plexippina, resulting in the reclassification of *Iranattus* Prószyński, 1992 (Marathe et al. 2024b) and the establishment of new genera (e.g. *Chuanattus* C. Wang et al., 2025; *Dianattus* C. Wang et al., 2025; *Ghatippus* Marathe & Maddison, 2024; *Gratianna* Caleb, 2025; *Phenasurya* Marathe & Maddison, 2024, and *Tenkana* Marathe et al. 2024) (Marathe et al. 2024a, 2024b, 2025; Caleb 2025; Wang et al. 2025). Continued taxonomic revisions, including the re-examination of type material, assessment of morphological variation, and resolution of synonymies, are essential for accurately documenting diversity and reconstructing the evolutionary history of this ecologically important subtribe.

In this study, we re-examined the type materials of seven species of Plexippina from China belonging to the genera *Evarcha*, *Pancorius*, *Thyene*, and *Telamonia*. Based on detailed morphological comparisons, three new synonyms are proposed.

## Material and methods

Specimens were stored in 75% ethanol. Vulvae were cleaned with lactic acid before examination and photography. Specimens were examined and measured with a Leica M205C stereomicroscope. Photos were taken with a Kuy Nice E3IS PM digital camera mounted on an Olympus BX53 compound microscope. Focused images were generated using Helicon Focus v. 7.6.1 and then modified in Adobe Photoshop CS6. The map was created by ArcMap v. 10.8.

The type material used in the current study is deposited in the College of Life Sciences, Hunan Normal University (**HUNNU**) in Changsha, China.

Abbreviations used in the text and figures are as follows: **a.s.l**., above sea level; **AS**, anterior chamber of spermatheca; **IZCAS**, Institute of Zoology, Chinese Academy of Sciences; **CD**, copulatory duct; **CO**, copulatory opening; **dRTA**, dorsal retrolateral tibial apophysis; **E**, embolus; **FD**, fertilisation duct; **juv**., juvenile; **P**, epigynal pocket; **PS**, posterior chamber of spermatheca; **RTA**, retrolateral tibial apophysis; **S**, spermatheca; **s. coll**., sine collectione; **SD**, sperm duct; **TA**, tegular apophysis; **TF**, tegular flap; **vRTA**, ventral retrolateral tibial apophysis; **VTA**, ventral tibial apophysis.

## Taxonomy

### Family Salticidae Blackwall, 1841

#### Genus Epeus

Taxon classificationAnimaliaAraneaeSalticidae

G.W. Peckham & E.G. Peckham, 1886

524B2BEF-2F0D-54AA-9722-69651A1BAA79

##### Type species.

*Epeus flavobilineatus* (Doleschall, 1859).

#### Epeus bicuspidatus

Taxon classificationAnimaliaAraneaeSalticidae

(Song, Gu & Chen, 1988)

7793B6B2-C97D-5378-A422-8A19F413909B

Figs 1, 2

Plexippodes bicuspidatus Song, Gu & Chen, 1988: 71, figs 6–8 (D♂).
Epeus bicuspidatus —Peng et al. 1993b: 48, figs 121–124 (♂, transferred from Plexippoides); Song et al. 1999: 508, fig. 291N–O (♂); Peng and Li 2002: 386, fig. 1A–D (♂); Yin et al. 2012: 1352, fig. 732a–d (♂); Meng et al. 2015: 147, figs 1, 2, 5, 6, 9–14 (♂, D♀); Peng 2020: 92, fig. 48a–d (♂); Vu et al. 2025: 3, figs 1A–F, 2A–E (♂♀); Hill 2025: 20, figs 12.1–2 (possible senior synonym of Epeus pengi).
Telamonia luxiensis Peng et al., 1998: 40, figs 13–15 (♀)—Yin et al. 2012: 1491, fig. 813a–c (♀); Peng 2020: 473, fig. 347a–c (♀). Syn. nov.

##### Type material of *Epeus bicuspidatus*.

***Holotpe*** ♂, China: • Hainan Province: Mt Jianfeng, 25 Jun. 1981, s. coll. ***Paratypes*** • 6♂♂, Mt jianfeng, 25 Jun. 1981, s. coll., not examined.

##### Type material of *Telamonia luxiensis*.

***Holotype*** ♀ (P-174), China: • Hunan Province: Luxi County, 15 Aug. 1994, H.M. Yan leg., stored at HUNNU, examined.

##### Diagnosis.

See Peng and Li (2002).

##### Redescription.

**Figure 1.**
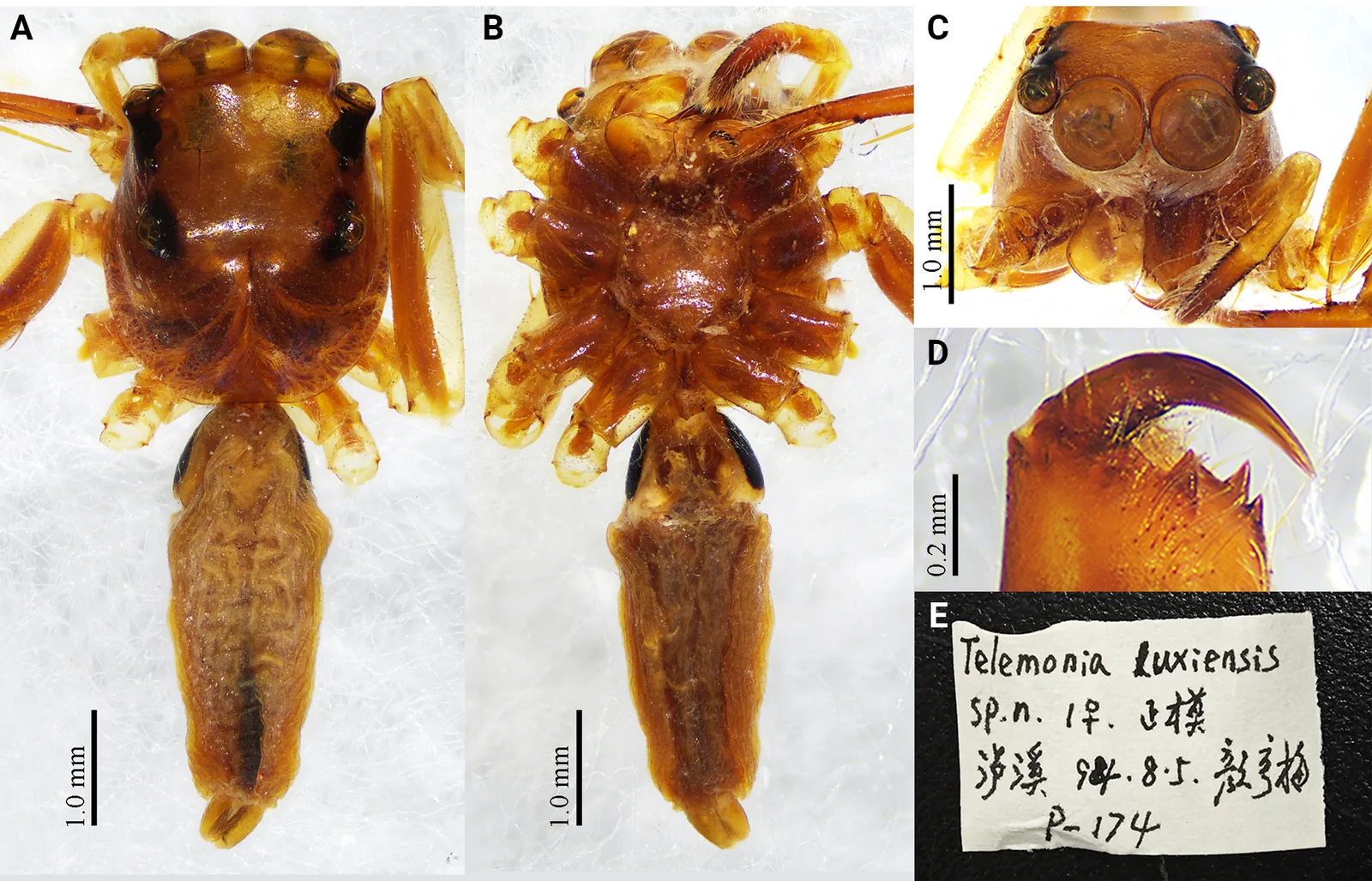
*Epeus bicuspidatus* (Song, Gu & Chen, 1988), holotype female of *Telamonia luxiensis*. **A**. Habitus, dorsal view; **B**. Ditto, ventral view; **C**. Ditto, anterior view; **D**. Chelicera, posterior view; **E**. Original label.

**Figure 2.**
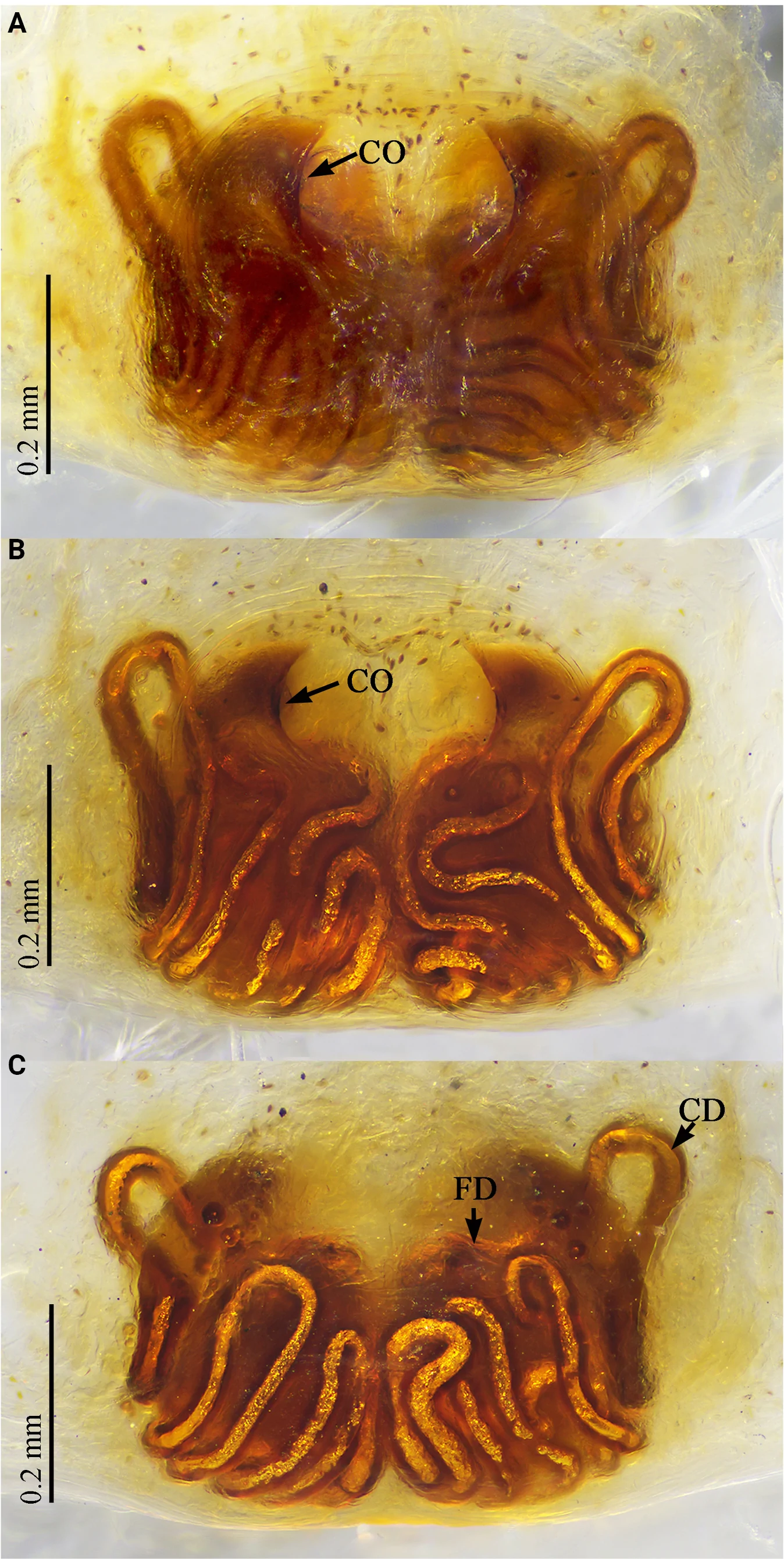
*Epeus bicuspidatus* (Song, Gu & Chen, 1988), holotype female of *Telamonia luxiensis*, genitalia. **A, B**. Epigyne, ventral view; **C**. Vulva, dorsal view. Abbreviations: CD, copulatory duct; CO, copulatory opening; FD, fertilization duct.

Detail description see Peng and Li (2002) and Meng et al. (2015). Photographs of the type of *T. luxiensis* are provided in Figs 1, 2.

##### Distribution.

China (Guizhou, Hunan, Hainan, Guangxi); Vietnam.

##### Remarks.

*Telamonia luxiensis* was described by Peng et al. (1998) based on a single female holotype from Luxi, Hunan, China. *Epeus bicuspidatus* was originally described by Song et al. (1988) based on the male holotype from Mt Jianfeng, Hainan, China, and its female was later described by Meng et al. (2015) based on specimens from Fanjingshan, Guizhou, China. The type localities of these two species are geographically close and the female genitalia have considerable morphological resemblance, including a shallow epigynal depression, inward-facing copulatory openings, and thin, strongly coiled copulatory ducts (Peng et al. 1998: figs 13–15; Meng et al. 2015: figs 11–14; Vu et al. 2025: fig. 1E, F). Therefore, *T. luxiensis* is considered to be a junior synonym of *E. bicuspidatus*.

#### Genus Evarcha

Taxon classificationAnimaliaAraneaeSalticidae

Simon, 1902

D8B9FC9D-24AC-5F09-ACDE-7F0F6C78BCAF

##### Type species.

*Araneus falcatus* Clerck, 1757.

#### Evarcha wulingensis

Taxon classificationAnimaliaAraneaeSalticidae

Peng, Xie & Kim, 1993

96D61FE2-098D-5A3C-B93E-96B1642857C8

Figs 3, 4

Evarcha wulingensis Peng et al., 1993a: 12, figs 30–34 (D♂)—Song et al. 1999: 511, fig. 296J (♂); Yin et al. 2012: 1367, fig. 742a–e (♂); Prószyński 2018: 148, fig. 9F (♂); Peng 2020: 143, fig. 91a–e (♂).

##### Type material.

***Holotype*** ♂, China: • Hainan Province: Wulingyuan, 25 Sep. 1988, X.J. Peng leg., stored at HUNNU, examined.

##### Diagnosis.

See Peng et al. (1993a).

##### Redescription.

Male (holotype) measurements: see Peng et al. (1993a).

**Figure 3.**
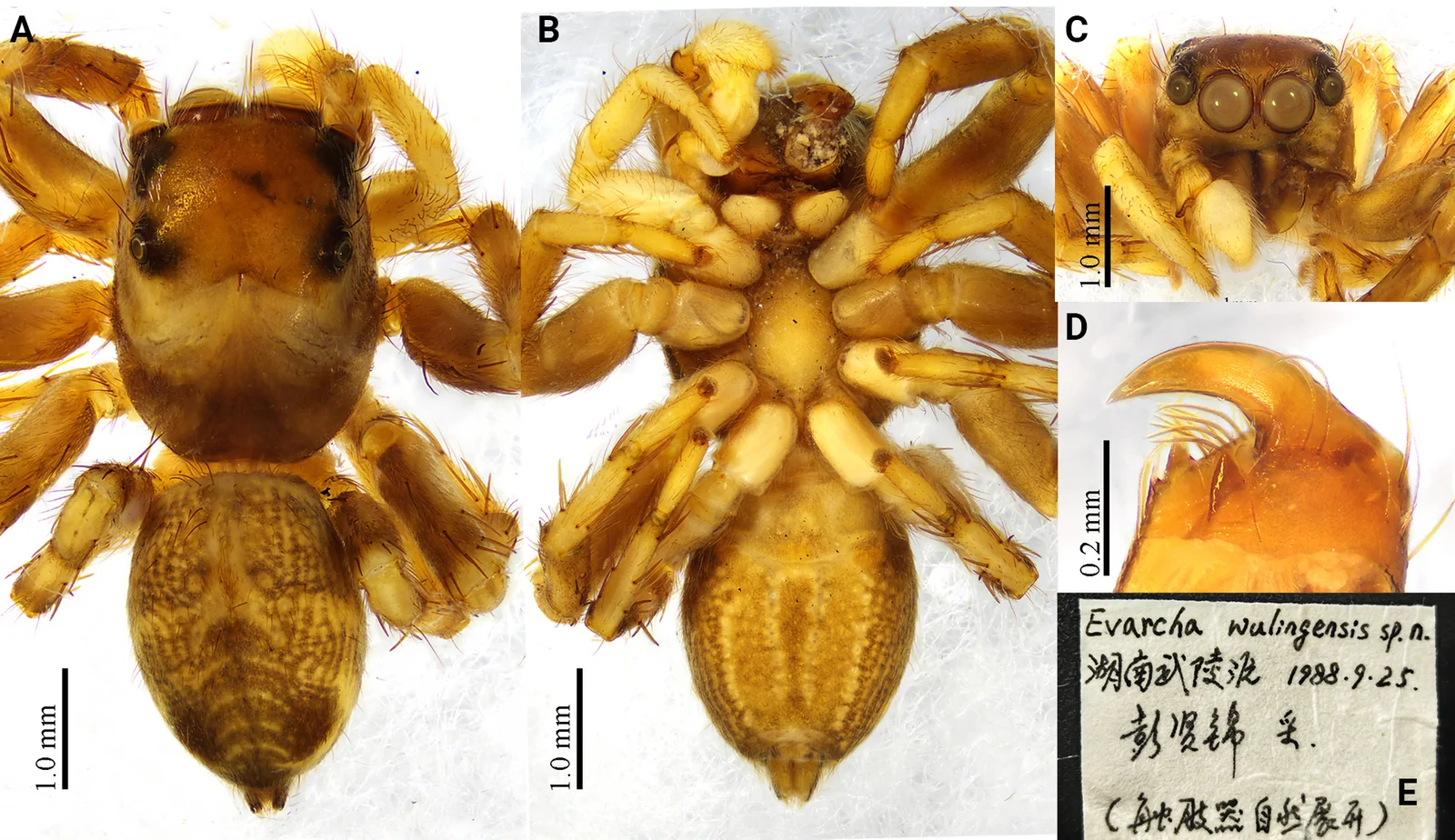
*Evarcha wulingensis* Peng, Xie & Kim, 1993, holotype male. **A**. Habitus, dorsal view; **B**. Ditto, ventral view; **C**. Ditto, anterior view; **D**. Chelicera, posterior view; **E**. Original label.

**Male** (Fig. 3A–D). Carapace longer than wide, yellow-brown, with yellow anterior area on thoracic part; cervical and radial grooves visible; fovea dark, longitudinal. Chelicerae yellow-brown, with two promarginal and one retromarginal teeth. Endites yellow, distally broadened. Labium concolourous with endites. Sternum longer than wide, yellow. Legs yellow-brown. Abdomen oval, with five yellow, chevron-shaped, posterior stripes. Venter yellow, with straight, longitudinal, serrated stripes. Spinnerets yellowish brown.

**Figure 4.**
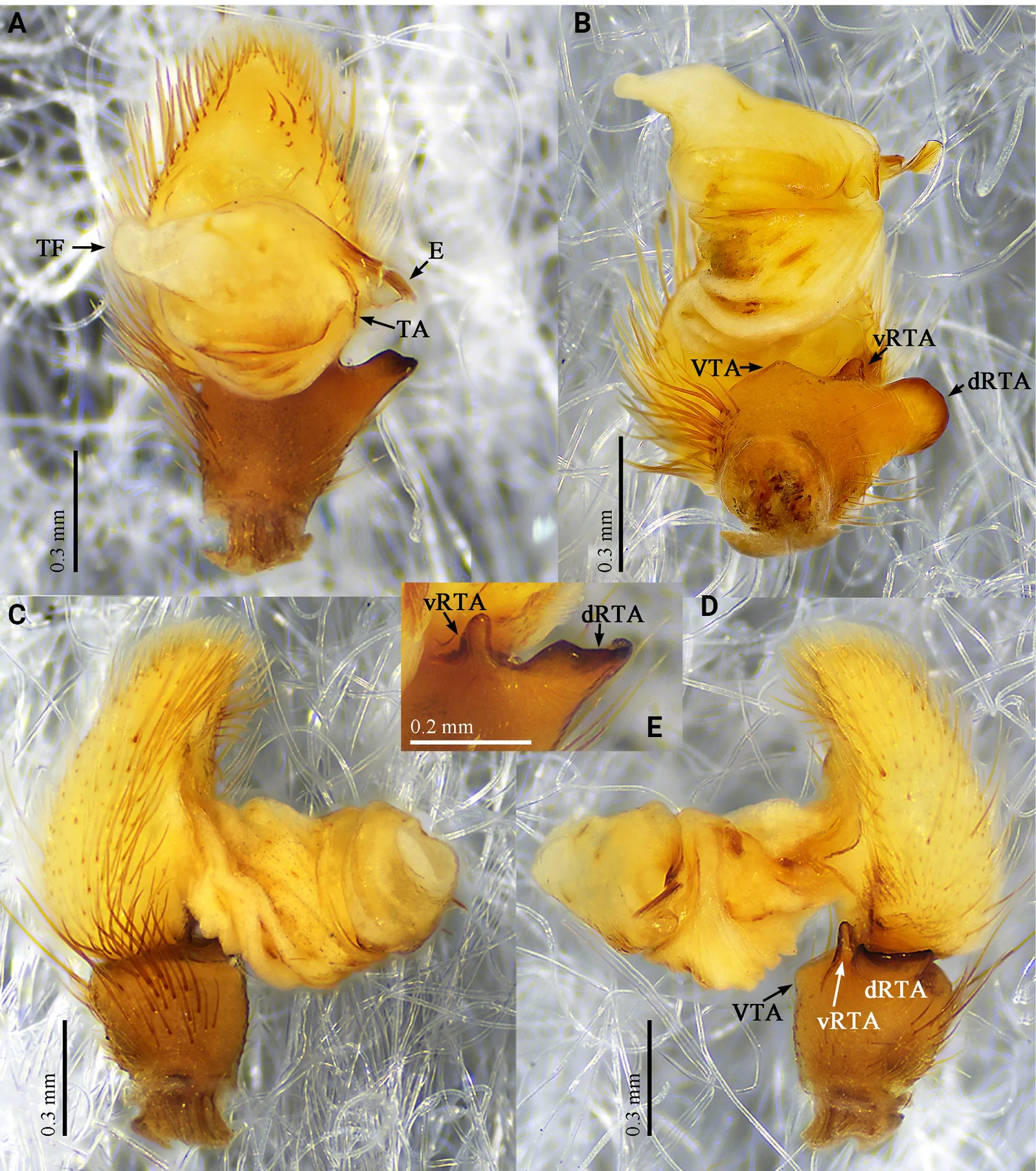
*Evarcha wulingensis* Peng, Xie & Kim, 1993, palp of male holotype. **A**. Palp, ventral view; **B**. Ditto, posterior view; **C**. Ditto, prolateral view; **D**. Ditto, retrolateral view; **E**. Retrolateral tibial apophysis, ventral view. Abbreviations: dRTA, dorsal retrolateral tibial apophysis; E, embolus; TA, tegular apophysis; TF, tegular flap; vRTA, ventral retrolateral tibial apophysis; VTA, ventral tibial apophysis.

***Palp*** (Fig. 4A–E). Tibia with three apophyses: dRTA wide and short; vRTA finger-like, with blunt tip; VTA shortest. Tegulum flat, with a conical TF, TA triangular with a sharp tip, situated near embolus. Embolus sclerotised, 1/2 length of tegulum.

**Female**. Unknown.

##### Distribution.

China (Hunan).

##### Notes.

In 1988, “Hunan Wulingyuan” was established as a county-level district under Dayong City. After Dayong was renamed Zhangjiajie in 1994, the area became today’s Zhangjiajie Wulingyuan District encompassing the renowned Wulingyuan Scenic Area.

#### Genus Pancorius

Taxon classificationAnimaliaAraneaeSalticidae

Simon, 1902

B0A603F6-D63F-55BB-82A2-004691C7E8C1

##### Type species.

*Ergane dentichelis* Simon, 1899.

#### Pancorius goulufengensis

Taxon classificationAnimaliaAraneaeSalticidae

Peng, Yin, Yan & Kim, 1998

15ACC7D2-4EBD-50DB-AC99-8B079A4248E8

Figs 5, 6

Pancorius goulufengensis Peng et al., 1998: 37, figs 4–6 (♀)—Yin et al. 2012: 1420, fig. 773a–c (♀); Peng 2020: 270, fig. 190a–c (♀).

##### Type material of *Pancorius goulufengensis*.

***Holotype*** ♀ (P-168), China: • Hunan Province: Hengyang City, Goulufengensis, 2 Aug. 1997, X.J. Peng leg., stored at HUNNU, examined.

##### Other material examined.

• 1♀ (HNU-YMS-2505-A), China: Hunan Province: Yongzhou City, Shuangpai County, Chashanluo, 26.0330, 111.9200, 971 m a.s.l., 25 Aug. 2025, X.J. Peng, S.L. Wang, Z.Y. Chen, F.Q. Wu, W.L. Yan leg., stored at HUNNU.

##### Diagnosis.

See Peng et al. (1998).

##### Redescription.

Female (holotype) measurements: see Peng et al. (1998).

**Figure 5.**
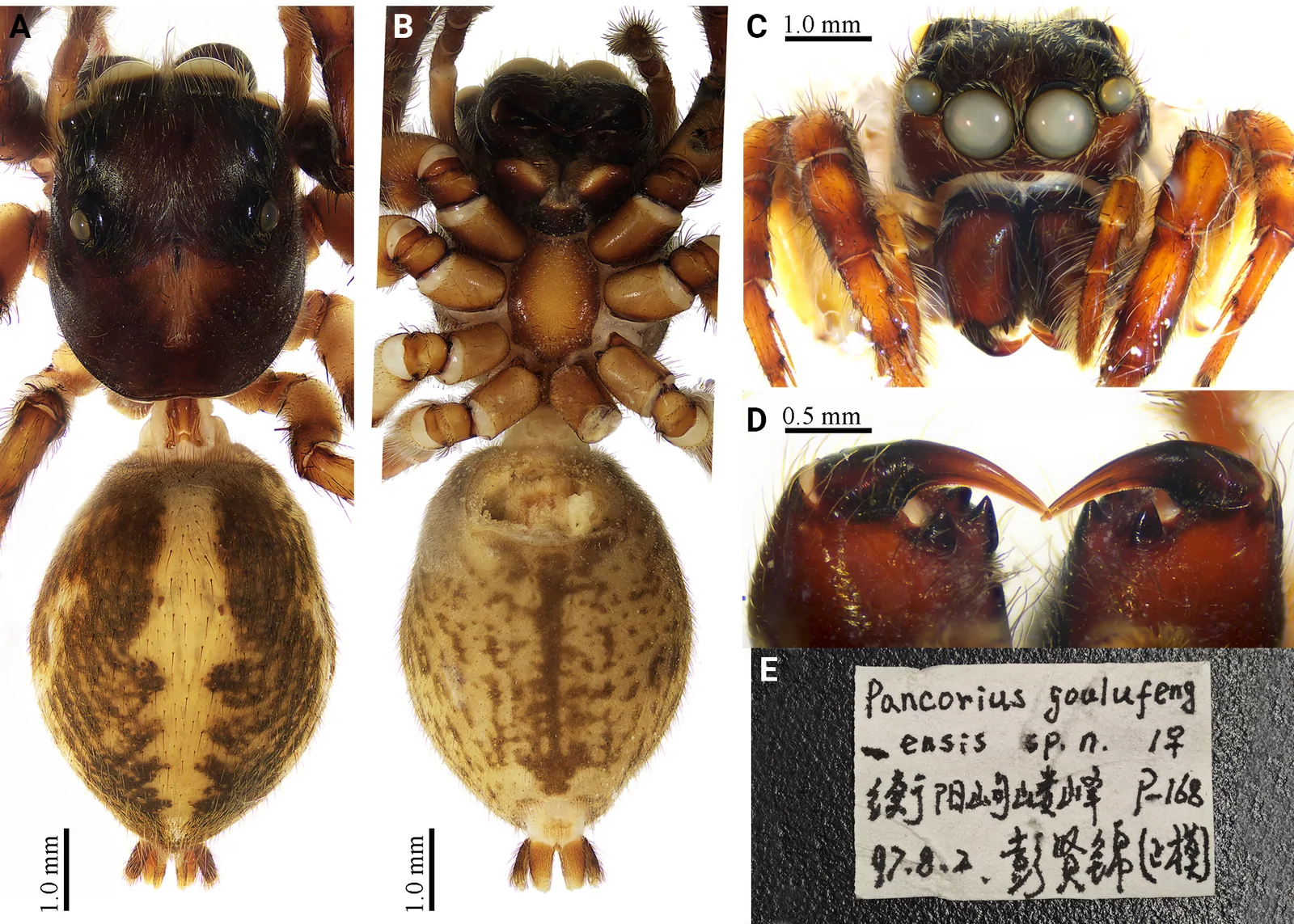
*Pancorius goulufengensis* Peng, Yin, Yan & Kim, 1998, holotype female. **A**. Habitus, dorsal view; **B**. Ditto, ventral view; **C**. Ditto, anterior view; **D**. Chelicerae, posterior view; **E**. Original label.

**Female** (Fig. 5A–D). Carapace dark brown, with an irregular, reddish-brown area on posterior part; fovea reddish brown, longitudinal. Chelicerae dark-reddish brown, with two promarginal teeth and one retromarginal tooth. Endites dark brown, paler on inner margins, distally broadened. Labium concolourous with endites. Sternum reddish brown to brown, shield-shaped, longer than wide. Abdomen oval, with dorsum brown, bearing a wide, longitudinal, median light band with posteromedian serrated margins; venter yellow, with brown longitudinal, discontinuous, dark patches. Spinnerets yellowish brown.

**Figure 6.**
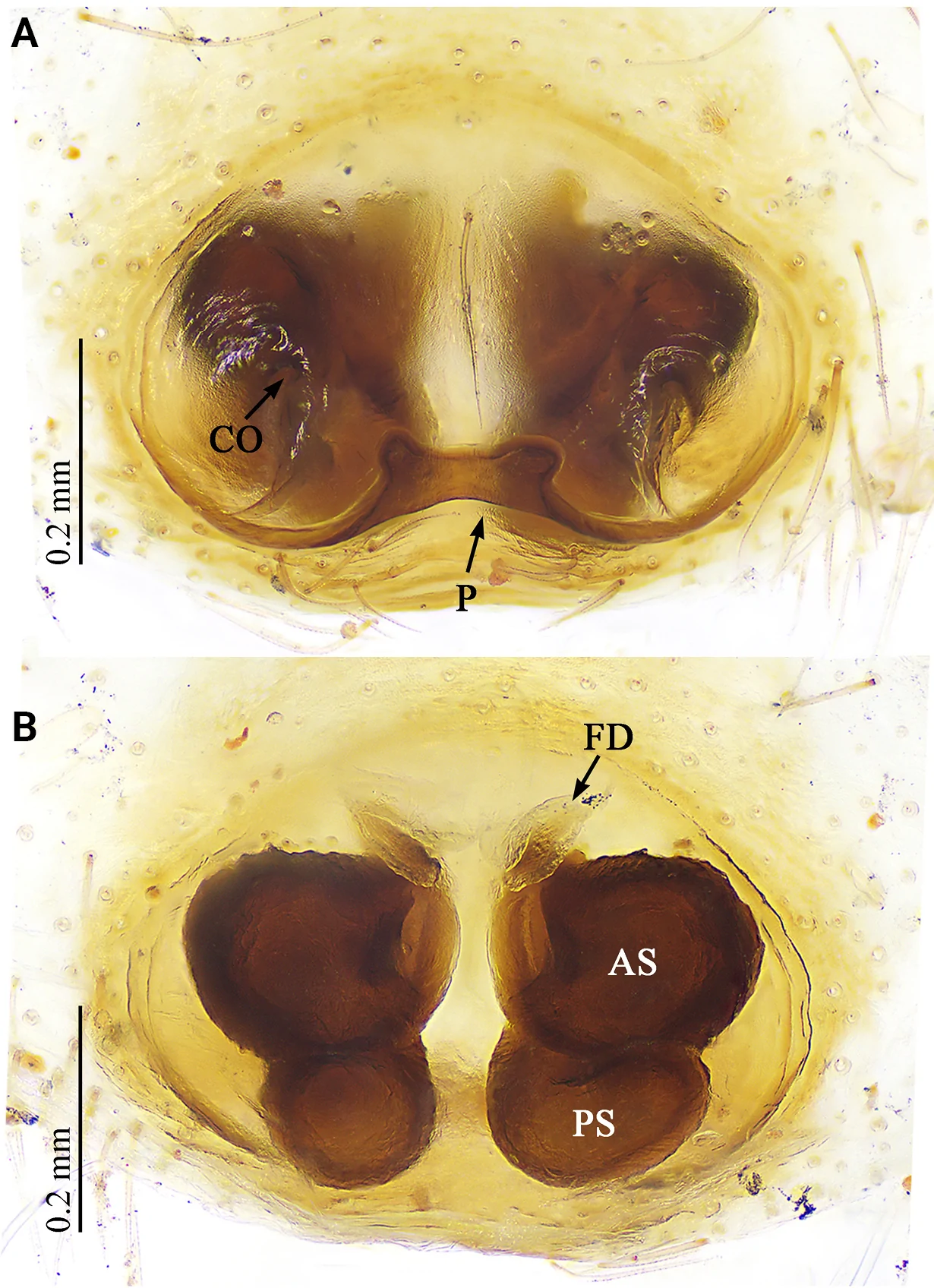
*Pancorius goulufengensis* Peng, Yin, Yan & Kim, 1998, genitalia of female holotype. **A**. Epigyne, ventral view; **B**. Vulva, dorsal view. Abbreviations: AS, anterior chamber of spermatheca; CO, copulatory opening; P, epigynal pocket; PS, posterior chamber of spermatheca; FD, fertilization duct.

***Epigyne*** (Fig. 6A, B). Epigynal plate wider than long; copulatory openings slit-shaped; M-shaped epigynal pocket situated medially; copulatory ducts invisible; spermathecae highly sclerotised, with two chambers, anterior one bean-shaped, left AS bigger than PS in dorsal view, the other side AS as wide as elongate tubiform PS; fertilisation ducts anterolaterally extended.

**Male**. Unknown.

##### Distribution.

China (Hunan).

#### Genus Ptocasius

Taxon classificationAnimaliaAraneaeSalticidae

Simon, 1885

07AF8388-14D1-5852-98EF-8D3E34246984

##### Type species.

*Ptocasius weyersi* Simon, 1885.

#### Ptocasius montiformis

Taxon classificationAnimaliaAraneaeSalticidae

Song, 1991

2E673958-3547-5F1E-BB8D-AA35680F7194

Figs 7, 8

Ptocasius montiformis Song, 1991: 163, fig. 1A–D (D♀)—Song et al. 1999: 543, fig. 313T–U (♀); Peng 2020: 377, fig. 273a, b (♀); Lin and Li 2020: 123 (synonym of Evarcha digitata).
Thyene triangula Xie & Peng, 1995: 106, fig. 3A–E (D♂)—Song et al. 1999: 561, figs 321Q, 322I, 330J (♂); Peng 2020: 486, fig. 358a–e (♂). Syn. nov.
Evarcha digitata Peng & Li, 2002: 469, fig. 1A–D (♂)—Prószyński 2018: 155, fig. 15I (♂); Peng 2020: 127, fig. 77a–d (♂).

##### Type material of *Ptocasius montiformis*.

***Holotype*** ♀, China: • Yunnan Province: Mengla County, Menglun, 20 Jul. 1984, s. coll. ***Paratype*** • 1♀, Menglun, 17 Mar. 1984, s. coll. Specimens stored at IZCAS, examined.

##### Type material of *Thyene triangula*.

***Holotype*** ♂ (P-93), China: • Yunnan Province: Mengla County, 12 March 1981, J.F. Wang leg., stored at HUNNU, examined.

##### Type material of *Evarcha digitata*.

***Holotype*** ♂ (CG075), China: • Guangxi Province: Zhuang Autonomous Region, 23.4, 105.8, 1350 m a.s.l., 18 Jun. 2000, J. Chen leg., stored at IZCAS, not examined.

##### Diagnosis.

This species is similar to *P. paraweyersi* Cao & Li, 2016 (Cao et al. 2016: figs 38A–D, 39A–E) in having slender embolus and flat tegulum, but it can be distinguished by the following: 1) embolus originating at 12 o’clock in ventral view (Fig. 8A), but at two o’clock in *P. paraweyersi* (Cao et al. 2016: fig. 38C, D); 2) retrolateral tibial apophysis longer than tibia in retrolateral view (Fig. 6B), but shorter in *P. paraweyersi* (Cao et al. 2016: fig. 38B); 3) one M-shaped epigynal pocket in ventral view (Peng 2020: fig. 273a), but two epigynal pockets in *P. paraweyersi* (Cao et al. 2016: fig. 39A); and 4) highest point of copulatory duct above fertilisation ducts in dorsal view (Peng 2020: fig. 273b), but at the same level or slightly lower in *P. paraweyersi* (Cao et al. 2016: fig. 39B).

##### Redescription.

**Figure 7.**
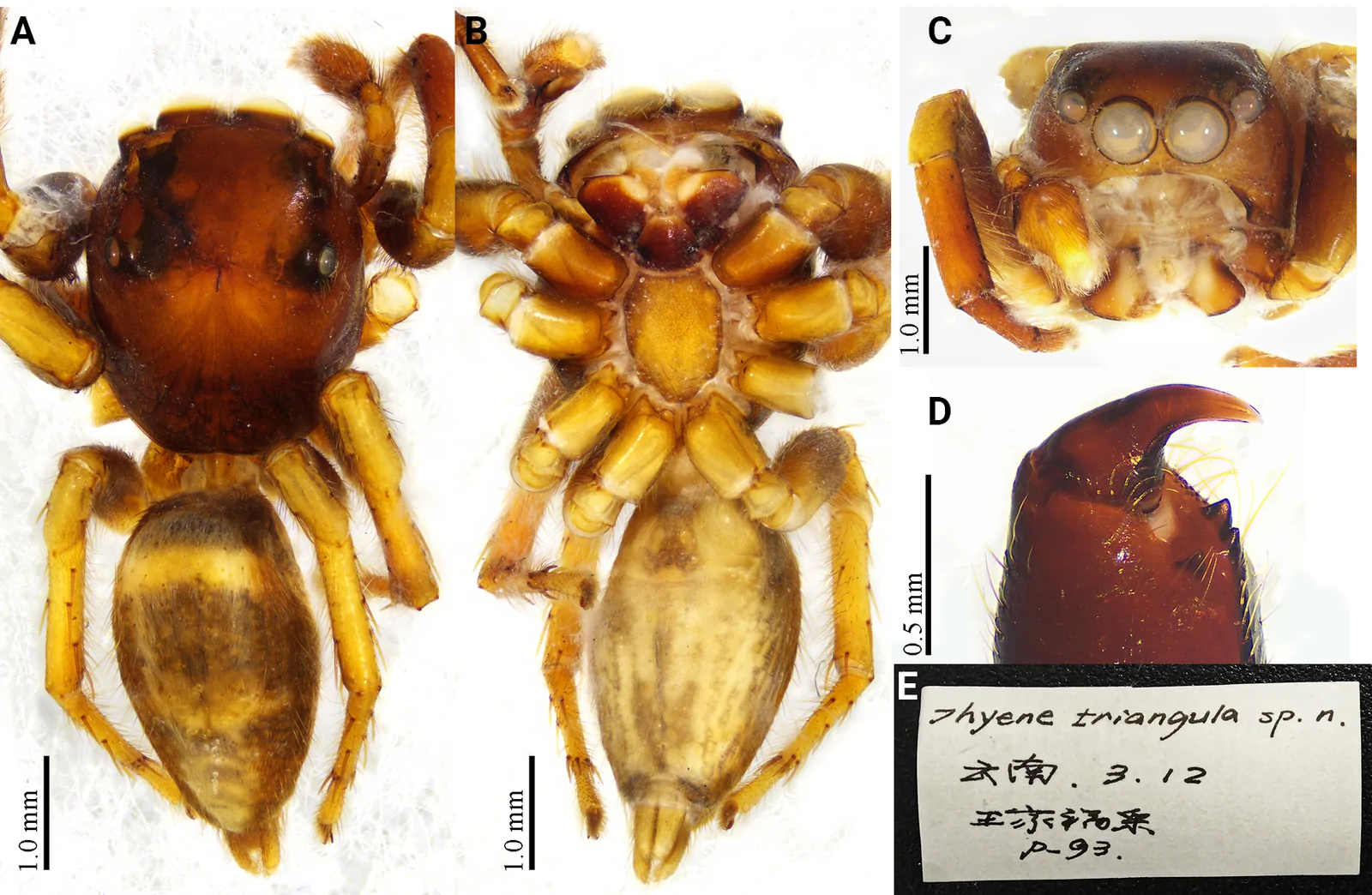
*Ptocasius montiformis* Song, 1991, holotype male of *Thyene triangula*. **A**. Habitus, dorsal view; **B**. Ditto, ventral view; **C**. Ditto, anterior view; **D**. Chelicera, posterior view; **E**. Original label.

**Figure 8.**
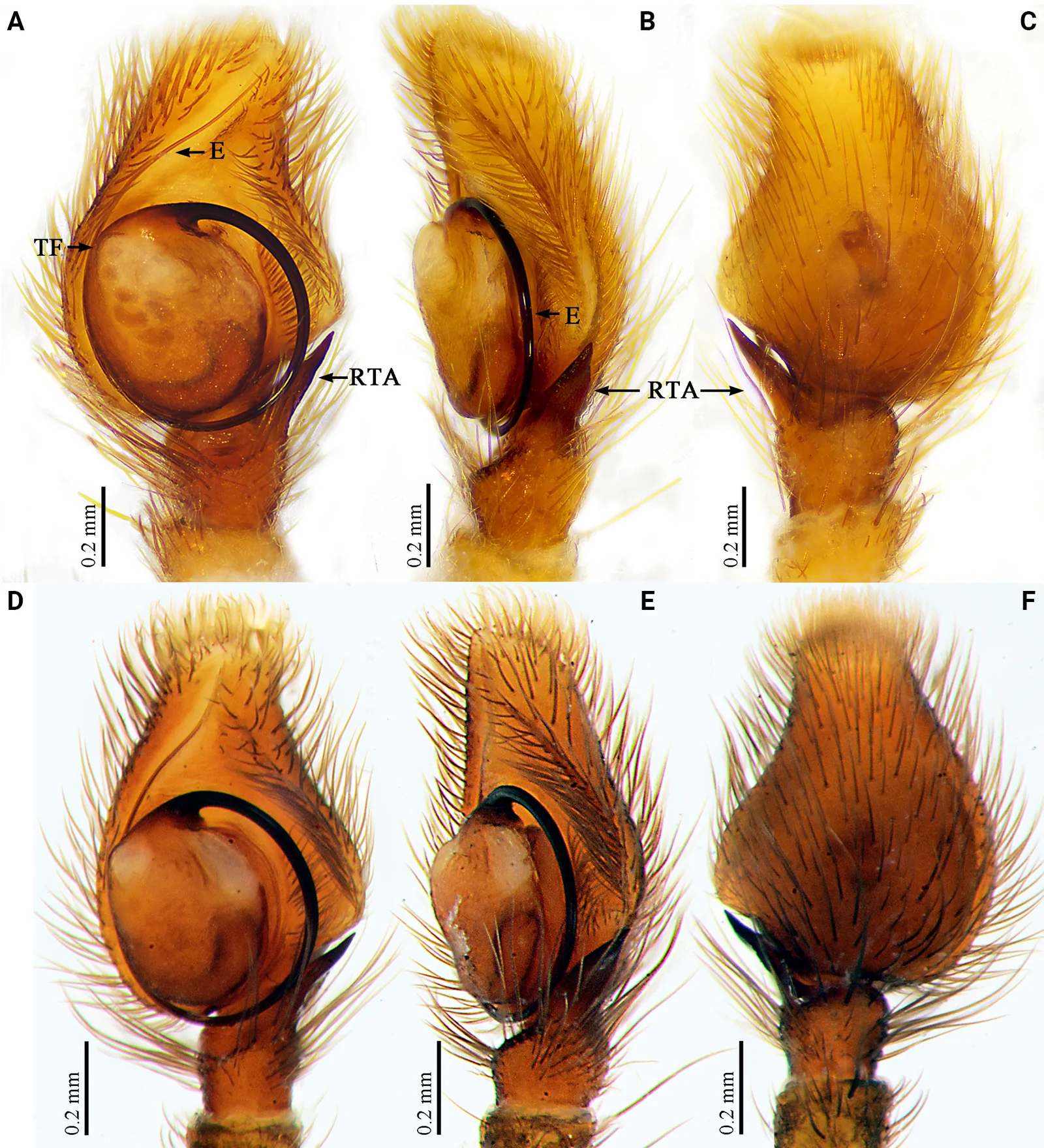
*Ptocasius montiformis* Song, 1991, holotype male of *Thyene triangula*, palp (**A–C**), holotype male of *Ptocasius montiformis*, palp (**D–F**). **A, D**. Palp, ventral view; **B, E**. Ditto, retrolateral view; **C, F**. Ditto, dorsal view. Abbreviations: E, embolus; RTA, retrolateral tibial apophysis; TF, tegular flap.

See Xie and Peng (1995). The types of *P. triangula* and *P. montiformis* are illustrated in Figs 7, 8 (*P. triangula*: Figs 7A–D, 8A–C; *P. montiformis*: Fig. 8D–F).

##### Distribution.

South Korea; China (Zhejiang, Fujian, Hubei, Hunan).

##### Remarks.

The type material of *T. triangula* was examined (Figs 7, 8A–C) and compared with high-quality images of *P. montiformis* obtained from the type-bearing institution (Institute of Zoology, Chinese Academy of Sciences, China) (Fig. 8D–F). No morphological difference was observed between the two species. Thus, *T. triangula* is considered to be a junior synonym of *P. montiformis*.

#### Genus Thyene

Taxon classificationAnimaliaAraneaeSalticidae

Simon, 1885

BA9C22F7-7652-5453-B64E-1FBC843CC964

##### Type species.

*Attus imperialis* Rossi, 1846.

#### Thyene orientalis

Taxon classificationAnimaliaAraneaeSalticidae

Żabka, 1985

7339F7DE-EC35-581A-AD89-C3E0A92DF73D

Figs 9, 10, 11, 12

Thyene orientalis Żabka, 1985: 454, figs 632–635 (D♂)—Peng et al. 1993b: 244, figs 869–873 (♂); Xie and Peng 1995: 107, fig. 6A–E (♂); Song and Li 1997: 442, fig. 53A–D (♂); Song et al. 1999: 562, figs 321M, N, 330H (♂); Yin et al. 2012: 1497, fig. 817a–d (♂); Ikeda,= 2013: 85, figs 16–25 (♂♀); Peng 2020: 484, fig. 356a–e (♂); Logunov 2021b: 1049, figs 136–151 (♂♀, synonym of Thyene radialis); Wang et al. 2023: 75, figs 50A–C, 51A–G (♂♀); Iwanaga et al. 2026: 83, figs A, B (♀).
Thyene radialis Xie & Peng, 1995: 105, fig. 2A–E (D♂)—Song et al. 1999: 563, figs 321O, P, 330L (♂); Yin et al. 2012: 1498, fig. 818a–e (♂); Peng 2020: 485, fig. 357a–e (♂).
Plexippoides zhangi Peng et al., 1998: 38, figs 7–9 (D♀)—Yin et al. 2012: 1440, fig. 785a–c (♀); Peng 2020: 334, fig. 240a–c (♀).
Thyene zhangi —Wang et al. 2023: 74 (transfer from Plexippoides). Syn. nov.

##### Type material of *Thyene orientalis*.

***Holotype*** ♂, ***paratypes*** • 1♂, 1 juv., Muséum national d’Histoire naturelle (MNHN) 22439, not examined.

##### Type material of *Thyene radialis*.

***Holotype*** ♂ (P-97), China: • Hunan Province: Chengbu County, 26 Jul. 1982, stored at HUNNU, J.F. Wang leg., examined.

##### Type material of *Thyene zhangi*.

***Holotype*** ♀ (P-175), China: • Hunan Province: Longshan County, Houyan, 9 Sep. 1995, stored at HUNNU, Y.J. Zhang leg., examined.

##### Diagnosis.

See Wang et al. (2023).

##### Redescription.

**Figure 9.**
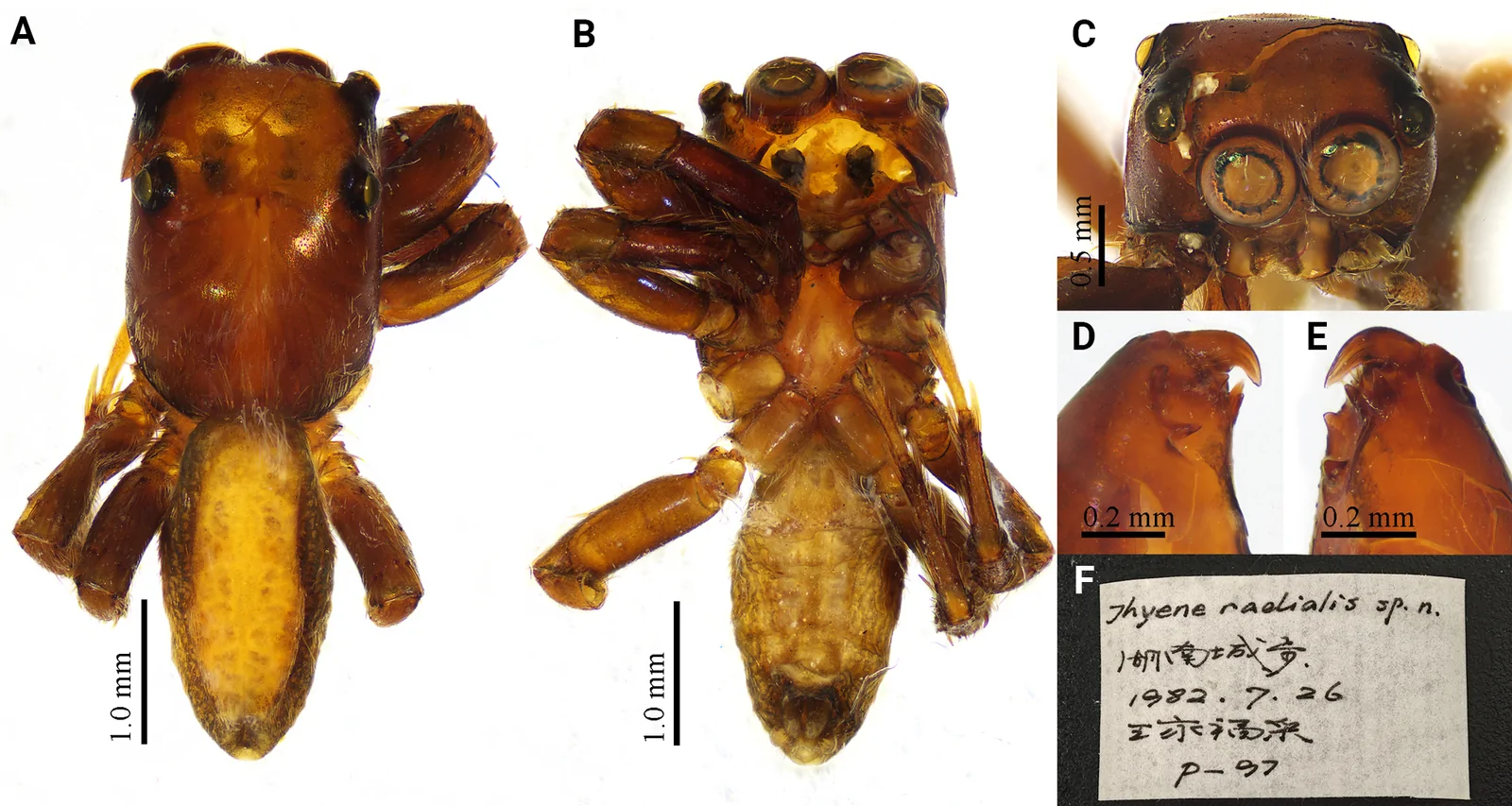
*Thyene orientalis* Żabka, 1985, holotype male of *Thyene radialis*. **A**. Habitus, dorsal view; **B**. Ditto, ventral view; **C**. Carapace, anterior view; **D**. Chelicera, posterior view; **E**. Ditto, anterior view; **F**. Original label.

**Figure 10.**
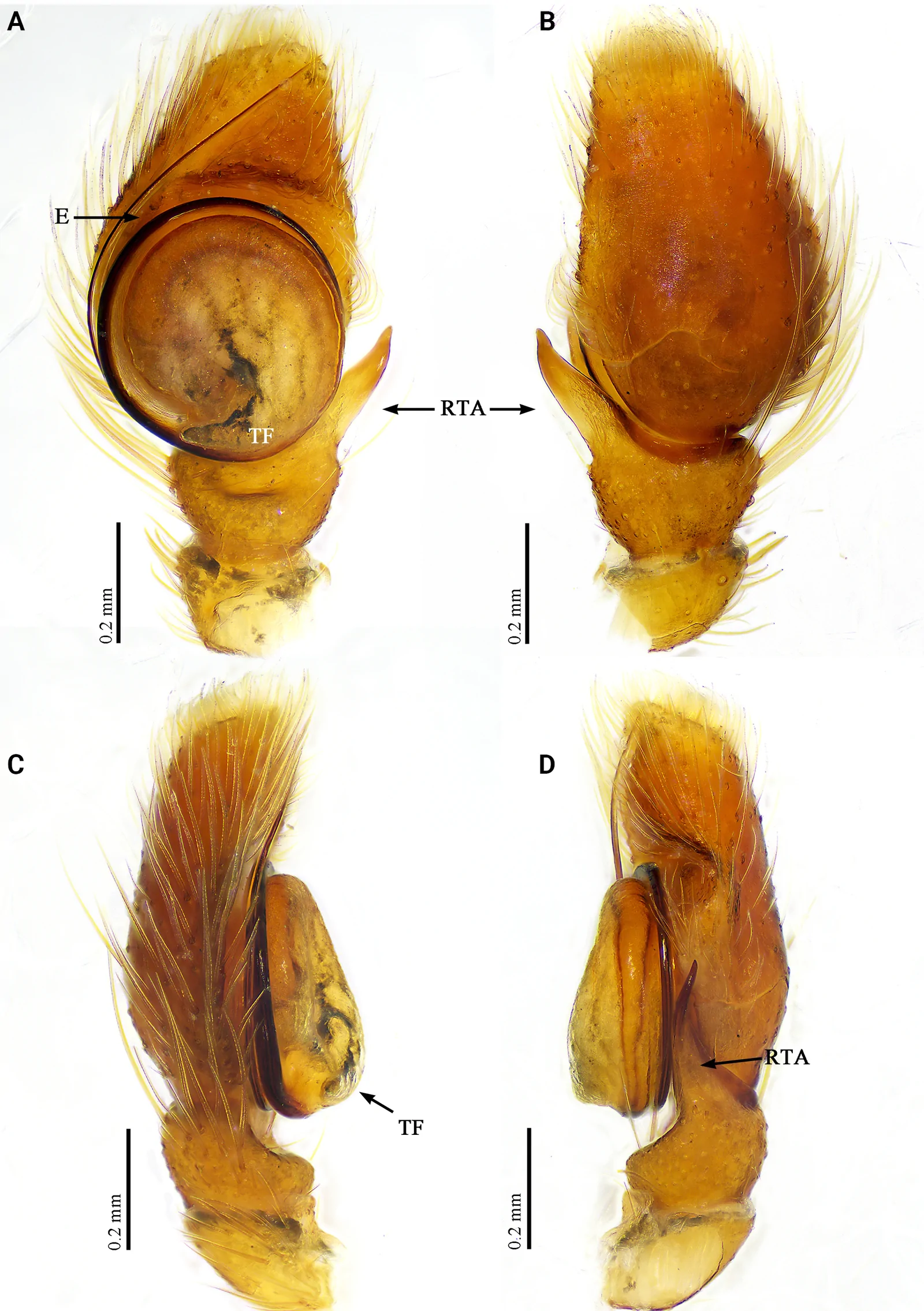
*Thyene orientalis* Żabka, 1985, holotype male of *Thyene radialis*, palp. **A**. Palp, ventral view; **B**. Ditto, dorsal view; **C**. Ditto, prolateral view; **D**. Ditto, retrolateral view. Abbreviations: E, embolus; RTA, retrolateral tibial apophysis; TF, tegular flap.

**Figure 11.**
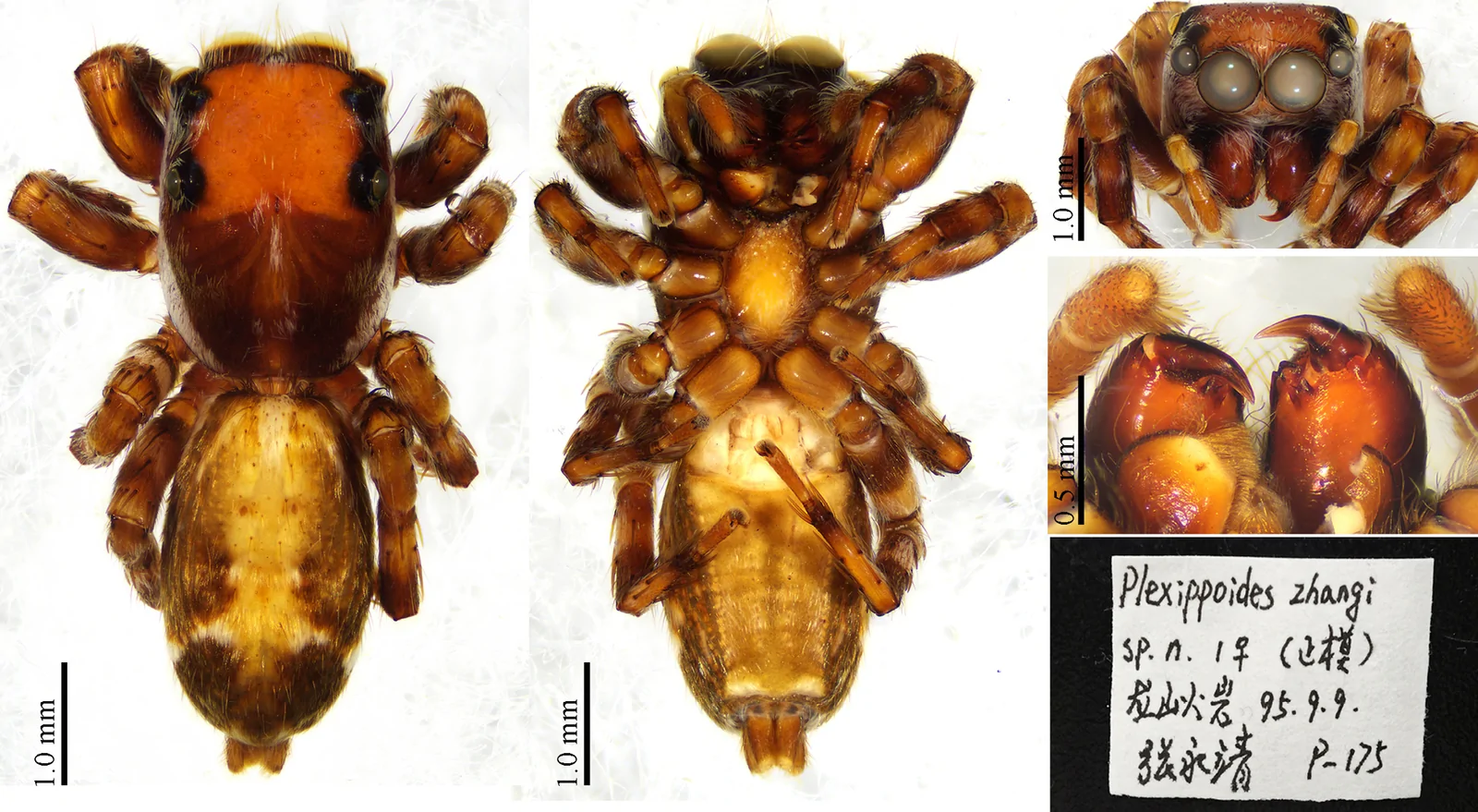
*Thyene orientalis* Żabka, 1985, holotype female of *Thyene zhangi*. **A**. Habitus, dorsal view; **B**. Ditto, ventral view; **C**. Ditto, anterior view; **D**. Chelicerae, posterior view; **E**. Original label.

**Figure 12.**
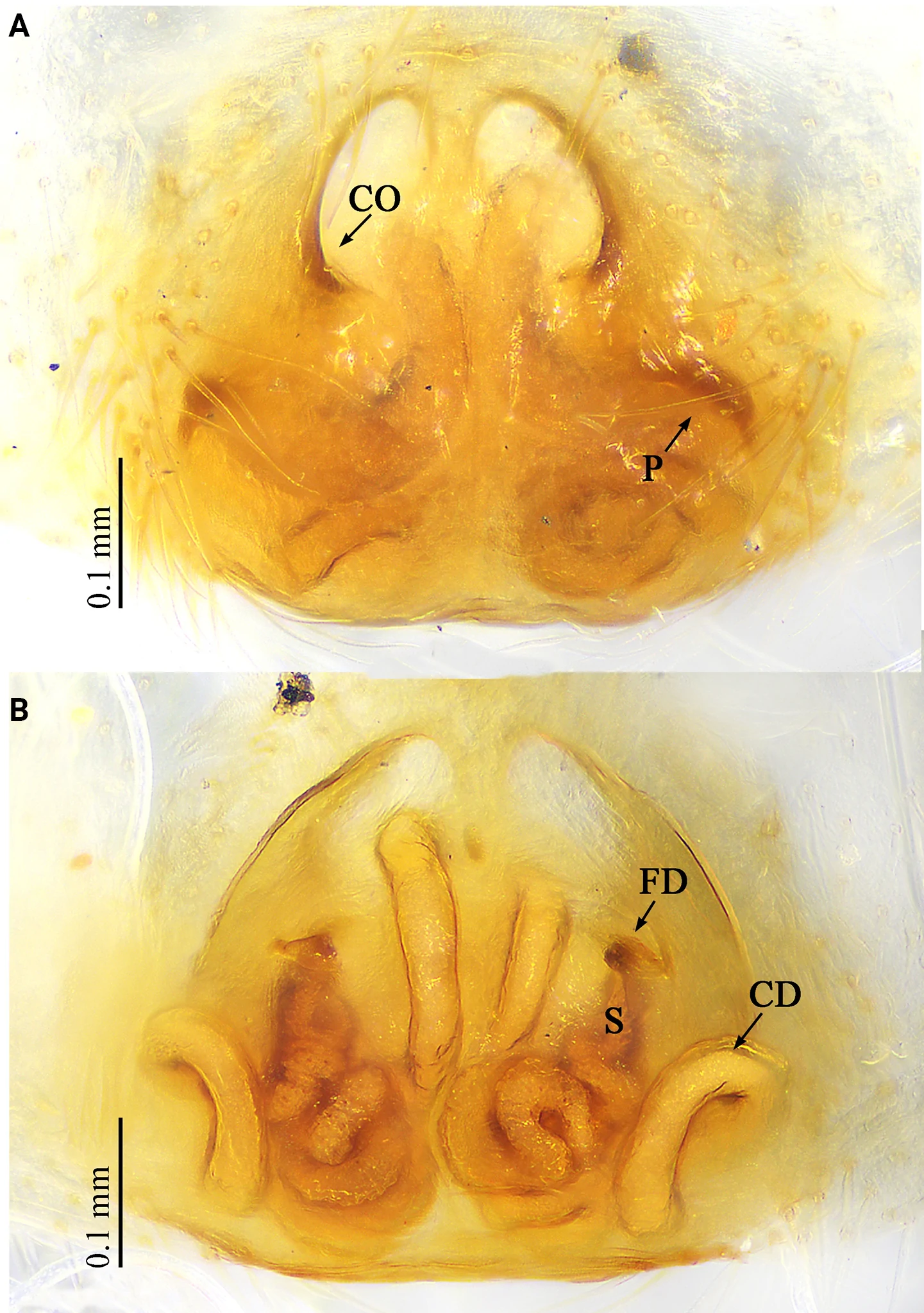
*Thyene orientalis* Żabka, 1985, holotype female of *Thyene zhangi*, genitalia. **A**. Epigyne, ventral view; **B**. Vulva, dorsal view. Abbreviations: CD, copulatory duct; CO, copulatory opening; FD, fertilization duct; P, epigynal pocket; S, spermatheca.

See Logunov (2021b) and Peng (2020). Photographs of the types of *Thyene radialis* are given in Figs 9, 10; photographs of the types of *Thyene zhangi* are given in Figs 11, 12.

##### Distribution.

Japan, China (Guangxi, Hunan), Vietnam.

##### Remarks.

*Thyene radialis* was treated as the synonymy of *T. orientalis* by Logunov (2021b). *Thyene radialis* closely resembles the original description and illustrations of *T. orientalis* in having the conical RTA, the slender embolus originating at the 6 o’clock position, and the linguiform TF (Fig. 10; Żabka 1985: figs 632, 633; Xie and Peng 1995: figs 2, 6). Although minor differences are observed, such as the origin of the TF, the thickness of the tegulum in retrolateral view, and the presence of three or four radial striae on the bulbus in *T. radialis* (Xie and Peng 1995: fig. 6B; Logunov 2021b: fig. 144; Wang et al. 2023: fig. 50B). These differences are considered to be the intraspecific individual variation.

Examination of the type material of *T. zhangi* found no morphological differences between it and *T. orientalis* (Fig. 12; Ikeda 2013: figs 24, 25; Wang et al. 2023: fig. 51), except for the bilateral asymmetry of the copulatory duct, which has been reported in *T. orientalis* by Iwanaga et al. (2026). Therefore, these differences are also interpreted as intraspecific individual variation rather than taxonomic distinctions, and *T. zhangi* considered herein as a junior synonym of *T. orientalis*.

In summary, *T. radialis* and *T. zhangi* are herein regarded as junior synonyms of *T. orientalis*.

#### Thyene yuxiensis

Taxon classificationAnimaliaAraneaeSalticidae

Xie & Peng, 1995

D1F422C7-D6C9-5C77-A82D-FF1D7F01A9D0

Figs 13, 14

Thyene yuxiensis Xie & Peng, 1995: 106, fig. 4A–E (D♂)—Song et al. 1999: 563, figs 322J, K, 330K (♂); Jastrzębski 2006: 2, figs 6–10 (♂); Peng 2020: 487, fig. 359a–e (♂); Logunov 2021a: 360, figs 42–43 (♂); Zhou et al. 2023: 540, figs 3A–D, 4A–E (♂, D♀).

##### Type material of *Thyene yuxiensis*.

***Holotype*** ♂ (P-92), China: • Yunnan Province: Yuxi County, 5 Jul. 1983, M.Y. Liu leg., stored at HUNNU, examined.

##### Diagnosis.

See Zhou et al. (2023).

##### Redescription.

**Male** (holotype) measurements: see Xie and Peng (1995).

**Figure 13.**
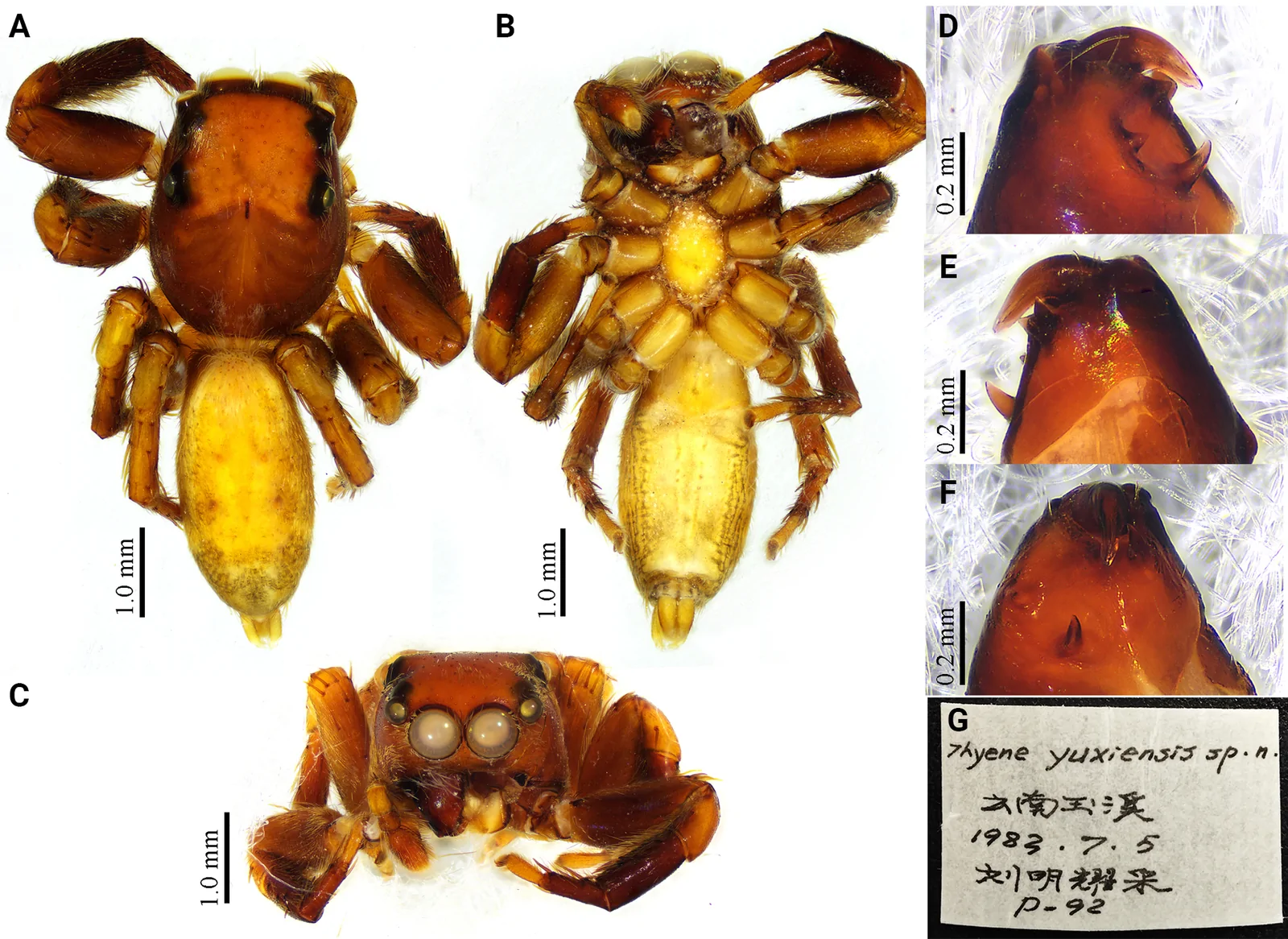
*Thyene yuxiensis* Xie & Peng, 1995, holotype male. **A**. Habitus, dorsal view; **B**. Ditto, ventral view; **C**. Ditto, anterior view; **D–F**. Chelicera; **D**. Posterior view; **E**. Anterior view; **F**. Lateral view; **G**. Original label.

***Colouration*** (Fig. 13A–F). Carapace longer than wide, red-brown, with dark lateral areas; cervical and radial grooves visible; fovea dark and longitudinal. Chelicerae red-brown, with three promarginal teeth and one retromarginal dichotomous tooth. Endites red-brown, yellow on inner margins, distally broadened. Labium concolourous with endites. Sternum longer than wide, yellow. Legs blackish red-brown. Abdomen elongate-oval, anterior margin with a tuft of grey-white bristles; three indistinct chevrons on posterior. Venter yellow, with three yellowish-brown, longitudinal striae, lateral margin with straight, serrated stripes. Spinnerets yellowish brown.

**Figure 14.**
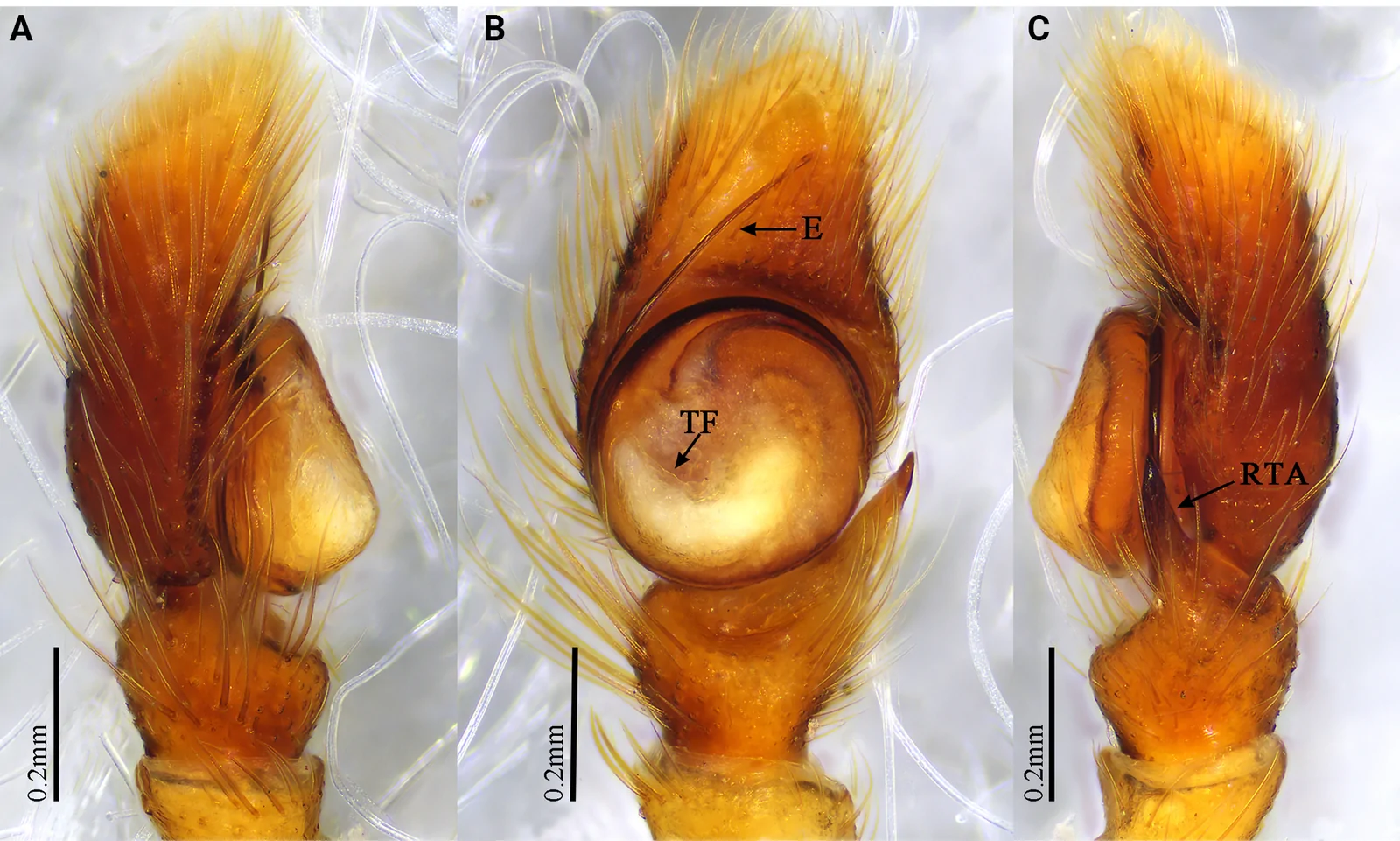
*Thyene yuxiensis* Xie & Peng, 1995, palp of male holotype. **A**. Palp, prolateral view; **B**. Ditto, ventral view; **C**. Ditto, retrolateral view. Abbreviations: E, embolus; RTA, retrolateral tibial apophysis; TF, tegular flap.

***Palp*** (Fig. 14A–C). Tibia wider than long. RTA tapered, as long as tibia, slightly curved and apically pointed in retrolateral view. Tegulum with a linguiform tegular flap. Embolus originating from upper part of tegulum and coiling around bulbus.

**Female**. See Zhou et al. (2023).

##### Distribution.

China (Hunan, Yunnan), Nepal.

##### Note.

Yuxi County was upgraded to the county-level Yuxi City in September, 1993, and was renamed as Hongta District of Yuxi Prefecture in 1998.

## Supplementary Material

XML Treatment for Epeus

XML Treatment for Epeus bicuspidatus

XML Treatment for Evarcha

XML Treatment for Evarcha wulingensis

XML Treatment for Pancorius

XML Treatment for Pancorius goulufengensis

XML Treatment for Ptocasius

XML Treatment for Ptocasius montiformis

XML Treatment for Thyene

XML Treatment for Thyene orientalis

XML Treatment for Thyene yuxiensis

## Additional information

### Artificial Intelligence (AI) use

The authors accept full responsibility for the content of the manuscript, including the disclosure of any use of AI.

No AI tools were used in the preparation of this manuscript.

### Author ORCIDs

Y. Zhou https://orcid.org/0009-0007-7540-1780

X. Peng https://orcid.org/0000-0002-2614-3910

### Data availability

All of the data that support the findings of this study are available in the main text.
